# Long non‐coding RNA MALAT1 regulates cell proliferation and apoptosis via miR-135b-5p/GPNMB axis in Parkinson’s disease cell model

**DOI:** 10.1186/s40659-021-00332-8

**Published:** 2021-03-16

**Authors:** Kefeng Lv, Yuhua Liu, Yanbing Zheng, Shaowen Dai, Peifeng Yin, Haifeng Miao

**Affiliations:** 1 Department of Neurology, Affiliated Dongguan People’s Hospital, Southern Medical University (Dongguan People’s Hospital), Guangdong Province 523059 Dongguan, People’s Republic of China; 2 Department of General Practice, Affiliated Dongguan People’s Hospital, Southern Medical University (Dongguan People’s Hospital), 3 South Wandao Road, Wanjiang District, 523059 Dongguan, Guangdong Province People’s Republic of China

**Keywords:** MALAT1, miR-135b-5p, GPNMB, Parkinson’s disease, Cell proliferation, Apoptosis

## Abstract

**Backgrounds:**

Parkinson’s disease (PD) is a common age-related neurodegenerative disorder worldwide. This research aimed to investigate the effects and mechanism underlying long non-coding RNA metastasis-associated lung adenocarcinoma transcript 1 (MALAT1) in PD.

**Methods:**

SK-N-SH and SK-N-BE cells were treated with MPP^+^ to establish the MPP^+^-stimulated cell model of PD, and MALAT1 expression was determined. Then, the effects of MALAT1 depletion on cell proliferation and apoptosis were determined in the MPP^+^-stimulated cell model of PD. Besides, the correlations between microRNA-135b-5p (miR-135b-5p) and MALAT1 or glycoprotein nonmetastatic melanoma protein B (GPNMB) in MPP^+^-stimulated cell model of PD were explored.

**Results:**

MALAT1 was increasingly expressed and downregulation of MALAT1 promoted cell proliferation while inhibited apoptosis in MPP^+^-stimulated cells. Besides, miR-135b-5p was a target of MALAT1 and directly targeted to GPNMB. Further investigation indicated that suppression of MALAT1 regulated cell proliferation and apoptosis by miR-135b-5p/GPNMB axis.

**Conclusion:**

Our findings reveal that MALAT1/miR-135b-5p/GPNMB axis regulated cell proliferation and apoptosis in MPP^+^-stimulated cell model of PD, providing a potential biomarker and therapeutic target for PD.

**Supplementary Information:**

The online version contains supplementary material available at 10.1186/s40659-021-00332-8.

## Highlights

1. MALAT1 was upregulated in MPP^+^-induced SK-N-SH and SK-N-BE cells.
2. MALAT1 depletion promoted cell proliferation and inhibited apoptosis in PD cell model.
3. MALAT1 upregulated GPNMN expression by sponging miR-135b-5p.
4. MALAT1 downregulation regulated MPP+-induced SK-N-SH and SK-N-BE cells proliferation and apoptosis by miR-135b-5p/GPNMB axis.

## Introduction

Parkinson’s disease (PD) is the second-most common neurodegenerative disease characterized by static tremors, bradykinesia, and cognitive dysfunction [1, 2]. According to epidemiological studies, approximately 1.7% of the Chinese population older than 65 years suffer from PD, which brings a heavy burden to patients’ families and society [3]. Mounting evidence suggested that PD is associated with multiple factors, such as inflammatory response, oxidative stress, and cell apoptosis [4]. There is numerous evidence suggesting that neuroinflammation is related to neurodegeneration in diverse neurological diseases [5]. Despite several alternative therapies used in clinical medicine, effective treatment for PD is still limited [6]. Thus, a better understanding of mechanism underlying PD and finding effective strategies for PD is urgently needed.

Long non-coding RNAs (lncRNAs), consist of more than 200 nucleotides, and have been reported to play essential roles in various human diseases [7–9]. A large mass of lncRNAs are found every year, and these lncRNAs are considered as “transcriptional noise” [10]. However, recent studies have disclosed the functions of lncRNAs in human diseases, including PD. For example, nuclear paraspeckle assembly transcript 1 (NEAT1) was obviously increased in the brain region of Huntington’s disease patients [11]. Metastasis-associated lung adenocarcinoma transcript 1 (MALAT1) is a well-known lncRNA, which has been proved to abnormal express in PD patients [12]. Moreover, MALAT1 has also been revealed that was closely related to multiple diseases [13]. Earlier studies have uncovered that MALAT1 regulated the development of 1-methyl-4-phenyl-1,2,3,6-tetrahydropyridine (MPTP)-stimulated PD cell model via modifying the expression of ɑ-synuclein [14]. These results highlighted the decisive role of MALAT1 in PD. However, the biological role of MALAT1 in the pathogenesis of PD is not fully understood.

MiRNAs, a class of RNAs, are deemed to be promising biomarkers in clinical therapy of neurodegenerative diseases via addressing central nervous system homeostasis [15–18]. According to earlier works, miRNAs exert their functions through binding to 3’-untranslatedregion (3’-UTR) of targeted mRNAs to degrade mRNA or inhibit the following translation process, thereby inhibiting the expression of the target gene [19, 20]. MiR-135b-5p, one of the common members of miRNAs, has been explored to participate in the drug resistance in breast cancer [21]. In addition, miR-135b-5p regulated cell metastasis and epithelial-mesenchymal transition (EMT) in gastric cancer by targeting CKLF-like MARVEL transmembrane domain containing 3 (CMTM3) [22]. Interestingly, a recent study suggested that miR-135b exerted a protective role in PD through suppressing pyroptosis [23]. Nevertheless, the role of miR-135b-5p in PD needs further investigation.

Glycoprotein non-metastatic melanoma protein B (GPNMB), a glycoprotein observed upon tissue damage and inflammation, the transcription of which is modulated by a microphthalmia-associated transcription factor (MITF) [24, 25]. Moreover, previous studies have shown that GPNMB level was selectively increased in PD patients [26, 27]. However, it is not unclear whether GPNMB was involved in MALAT1-mediated regulation in PD pathology.

As MPP^+^ could cause oxidative stress and mitochondrial dysfunction, thus injure dopaminergic neurons, leading to PD pathophysiology [28, 29], SK-N-SH and SK-N-BE cells were treated with MPP^+^ to establish the MPP^+^-stimulated cell model of PD, and we aimed to investigate the biological role and mechanism underlying MALAT1 in PD.

## Materials and methods

### Cell culture and treatment

Human neuroblastoma cells of SK-N-SH and SK-N-BE were purchased from American Type Culture Collection (ATCC, Manassas, VA, USA). Cells were cultured in Dulbecco’s modified Eagle medium (DMEM; Gibco, Carlsbad, CA, USA) supplemented with 10% fetal bovine serum (FBS; Gibco) and 1 × penicillin & streptomycin (Gibco, the final concentration of 100 U/mL penicillin and 100 µg/mL streptomycin). SK-N-SH or SK-N-BE cells re-suspended in complete medium were incubated under a humid atmosphere of 5% CO_2_ at 37 °C.

1-methyl-4-phenyl pyridinium (MPP^+^; Sigma-Aldrich, Louis, MO, USA) was used to establish PD cell model. To select the most appropriate concentration of MPP^+^, 0, 0.5, 1, 2, or 3 mM MPP^+^ were added into SK-N-SH and SK-N-BE cells, respectively. 2 mM was selected as the corresponding concentration for subsequent assays. Besides, SK-N-SH or SK-N-BE cells were pretreated with 100 µM MPP^+^ for 24 h to establish the *in vitro* cell model for PD.

### Transient transfection

Small interfering RNAs (siRNA) against MALT1 (si-MALAT1-1, si-MALAT1-2 or si-MALAT1-3) and its negative control (NC) were constructed. Overexpression vectors of MALAT1 (pcDNA3.0-MALAT1) and GPNMB (pcDNA3.0-GPNMB) were constructed by using pcDNA3.0, and pcDNA3.0 empty vector was used as negative control (pcDNA3.0-control). Besides, miR-135b-5p mimic (miR-135b-5p) and inhibitor (miR-135b-5p-inhibitor), as well as relative controls (miR-control as mimic control and miR-NC as inhibitor control) were constructed. These vectors or oligonucleotides were totally synthesized in KeyGEN Biotech. Cell transfection was conducted using lipofectamine 2000 (Invitrogen) in accordance with manufacturer’s instructions. In brief, 1 × 10^6^ SK-N-SH and SK-N-BE cells were cultured on the 6-well plates and incubated to 70% confluence. Then the constructs were incubated with 10 µL lipofectamine 2000 reagent for 5 min at room temperature and incubated with cells for 24, 48 or 72 h, followed by treatment with 2 mM MPP^+^ for 24 h.

### Cell viability detection

3-(4,5-Dimethylthiazol-2-yl)-2,5-diphenyl-tetrazolium bromide(MTT) assay was conducted to expose the capacity of cell proliferation. 1 × 104 SK-N-SH and SK-N-BE cells were seeded into a 96-well plate. After transfection for 0, 24, 48, or 72 h, respectively, 10 µl MTT solution (APExBIO Technology, Austin, TX, USA) was supplemented into each well, and then cells were cultured for another 4 h in the incubator at 37 °C. Then, dimethyl sulfoxide (DMSO; Absin Bioscience, Shanghai, China) was added to dissolve formazan. The absorbance value at 490 nm was detected using a microplate reader (Bio-Rad Laboratories, Philadelphia, PA, USA).

### Quantitative real‐time polymerase chain reaction (qRT-PCR) assay

Total RNA was isolated and extracted from induced SK-N-SH and SK-N-BE cells by Trizol reagent (Invitrogen, Carlsbad, CA, USA) in accordance with the manufacturer’s instruction. First strand of complementary DNA (cDNA) was synthesized by utilizing Reverse Transcriptase M-MLV (Takara, Dalian, China). QRT-PCR was carried out to measure levels of MALAT1, miR-135b-5p and GPNMB via SYBR Green qPCR Master Mix (MedChemExpress, New Jersey, NJ, USA) reagent kit and ABI 7500 system (Applied Biosystems, Foster City, CA, USA) according to the manufacturer’s procedures. Relative levels of MALAT1, miR-135b-5p and GPNMB were calculated by 2−ΔΔCt method, glyceraldehyde-3-phosphate dehydrogenase (GAPDH for MALAT1 and GPNMB) or U6 (for miR-135b-5p) were used as the internal control. Primer sequences were listed: MALAT1 (forward: 5′-AAAGCAAGGTCTCCCCACAAG-3′; reverse: 5′-GGTCTGTGCTAGATCAAAAGGCA-3′); miR-135b-5p (forward: 5′-GGTATGGCTTTTCATTCCT-3′; reverse: 5′-CAGTGCGTGTCGTGGAGT-3′); GPNMB (forward: 5′-AAGTGAAAGATGTGTACGTGGTAACAG-3′; reverse: 5′-TCGGATGAATTTCGATCGTTCT-3′);GAPDH (forward: 5′-TGAACGGGAAGCTCACTGG-3′; reverse: 5′-TCCACCACCCTGTTGCTGTA-3′); U6 (forward: 5′-CTCGCTTCGGCAGCACA-3′; reverse: 5′-AACGCTTCACGAATTTGCGT-3′). The above sequences were synthesized in KeyGEN Biotech (Jiangsu, China).

### Flow cytometry for cell apoptosis

Annexin V-FITC-fluorescein isothiocyanate/propidium iodide (Annexin V-FITC/PI) apoptosis detection kit (TransGen Biotech, Beijing, China) was employed to determine cell apoptosis in accordance with the manufacturer’s instruction. In brief, 1 × 10^6^ SK-N-SH and SK-N-BE cells were collected and then resuspended with 1× binding buffer. Annexin V-FITC (5 µL) and PI (5 µL) were incubated with cell suspension for 15 min at room temperature in the dark. The apoptosis rate of SK-N-SH and SK-N-BE cells were assessed by a flow cytometry (FACS; BD Biosciences, San Jose, CA, USA) and analyzed with Flowjo software.

### Western blot

Total proteins were extracted from SK-N-SH and SK-N-BE cells using RIPA lysis buffer (Beyotime, Shanghai, China). Equal amount (20 µg) of total proteins were separated by gel electrophoresis with 12% SDS-polyacrylamide gel (SDS-PAGE), and then transfected onto the PVDF membranes (polyvinylidene fluoride; Millipore, Bedford, MA, USA). Subsequently, membranes were incubated with Tris-Buffered Saline Tween-20 (TBST; Solarbio, Beijing, China) supplemented with 5% bovine serum albumin for 1 h at room temperature. Then the membrane was cultivated overnight at 4 °C with primary antibodies purchased from Abcam (Abcam, Cambridge, MA, USA) which was diluted in TBST solution. The primary antibodies used in this research were shown as below: Cleaved-caspase 3 (Cleaved-casp-3; 1:500, ab13847), B-cell lymphoma-2 (Bcl-2; 1:1000, ab32124), Bcl-2-Associated X (Bax; 1:7000, ab32503), GPNMB (1:100, ab227695), PCNA (1: 1000, ab18197) and GAPDH (1:1000, ab8245). Subsequently, the membranes was washed with TBST for three times and then incubated with corresponding secondary antibody Goat Anti-Rabbit IgG H&L (1: 20,000, ab205718, Abcam) for 1 h at room temperature. Then the bands were visualized using High sensitivity ECL chemiluminescence detection kit (Vazyme, Nanjing, China) and quantified by Quantity One software version 4.6 (Bio-Rad Laboratories). GAPDH was used as the internal control.

### Dual‐luciferase reporter assay

StarBaseV3.0 was employed to predict the potential targets of MALAT1 and miR-135b-5p. The sequences of MALAT1 or GPNMB 3’ non-translated regions (3’UTR) containing wild-type or mutant miR-135b-5p binding sites were amplified and then inserted into the pmirGLO (Promega, Madison, WI, USA) vectors to construct the recombinant luciferase reporter vectors, named as MALAT1-WT and MALAT1-MUT or GPNMB-WT and GPNMB-MUT, respectively. Then miR-135b-5p mimic or miR-control were co-transfected into SK-N-SH and SK-N-BE cells with the constructed luciferase reporter vectors. 48 h upon transfection, relative luciferase activities were identified using the dual-luciferase reporter assay kit (Promega). Each sample was performed thrice, and firefly luciferase activity was normalized to Renilla luciferase activity.

### Statistical analysis

Statistical analysis was conducted by SPSS17.0. All data of three independent were presented as mean ± standard deviation (SD). The differences between the two groups were evaluated by Student’s *t*-test, and multiple statistical significance of the differences were analyzed by one-way ANOVA. P < 0.05 was considered as a significant difference.

## Results

### MALAT1 was upregulated in MPP^+^-stimulated SK-N-SH and SK-N-BE cells

**Fig. 1:**
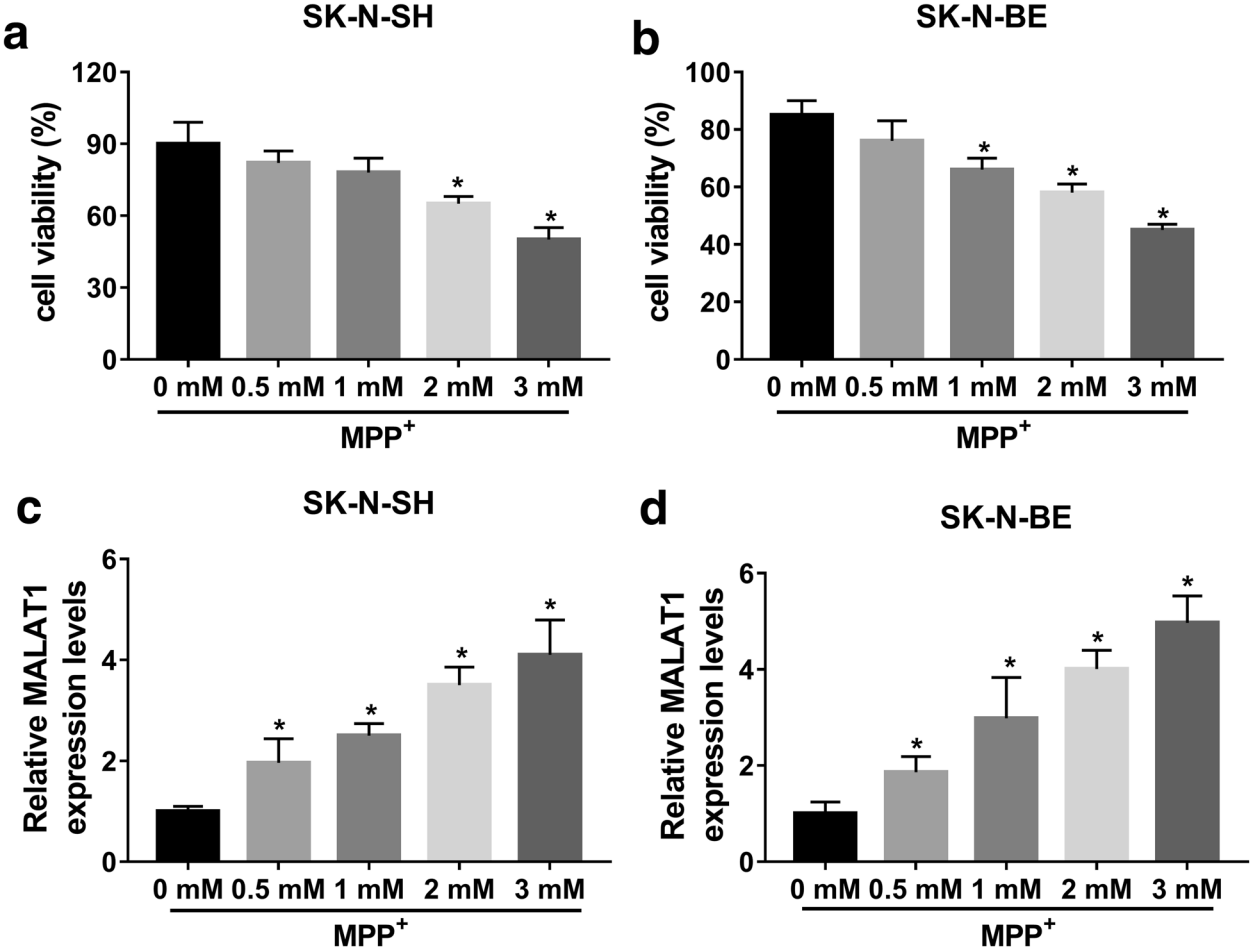
MALAT1 was overexpressed in MPP^+^-stimulated SK-N-SH and SK-N-BE cells. SK-N-SH and SK-N-BE cells were treated with different concentration of MPP^+^ (0 mM, 0.5 mM, 1 mM, 2 mM, or 3 mM). **a** and **b** Cell viability in MPP^+^- stimulated SK-N-SH and SK-N-BE cells were determined by MTT assay. **c** and **d** The expression of MALAT1 in MPP^+^- stimulated SK-N-SH and SK-N-BE cellswas measured by qRT-PCR. The experiments were repeated three times (n = 3). *P < 0.05, compared to untreated group

To investigate the expression of MALAT1 in PD, *in vitro* model of PD was established by MPP^+^-stimulated SK-N-SH and SK-N-BE cells. Firstly, SK-N-SH and SK-N-BE cells were cultured with different concentrations of MPP^+^ (0 mM, 0.5 mM, 1 mM, 2 mM, or 3 mM), cells with neuron-like morphology express synapse-specific proteins and display synaptic structures were chosen for further research (Additional file 1: Figure S1). MTT assay indicated that cell viability was significantly decreased in high concentration of MPP^+^-treated (2, and 3 mM) SK-N-SH and SK-N-BE cells (Fig. 1a and b), compared to untreated group. Besides, the level of MALAT1 was markedly increased in a concentration-dependent manner in SK-N-SH and SK-N-BE cells, especially in high levels of MPP^+^ treatment group (2, and 3 mM) (Fig. 1c and d). Therefore, 2 mM MPP^+^ was selected as the treatment conditions for subsequent experiments.

### MALAT1 knockdown contributed to cell proliferation, curbed apoptosis in MPP^+^-stimulated SK-N-SH and SK-N-BE cells

**Fig. 2:**
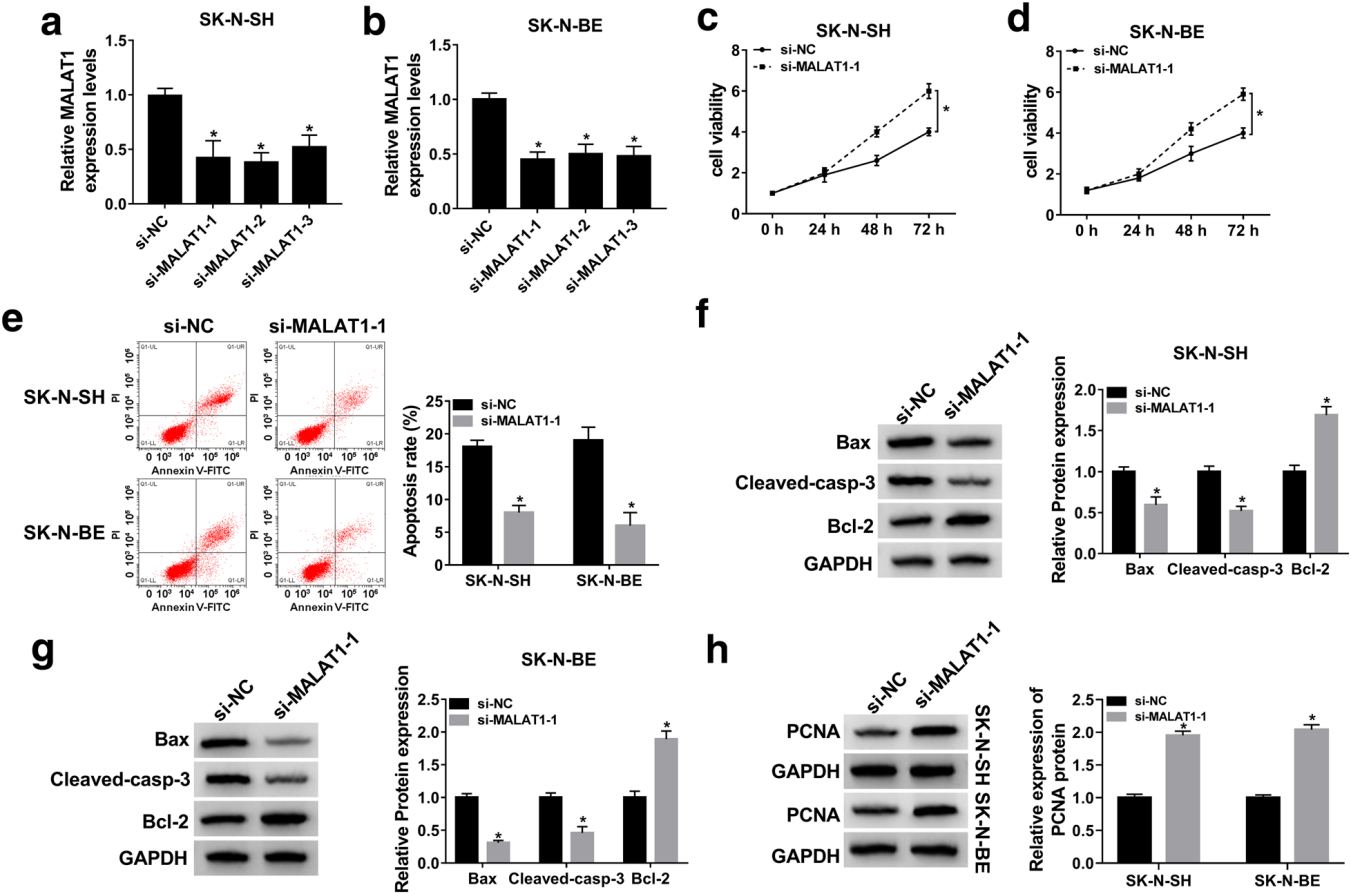
MALAT1 knockdown promoted cell proliferation but inhibited apoptosis in MPP^+^-stimulated cell model of PD. **a**, **b** MPP+-stimulated SK-N-SH and SK-N-BE cells were transfected with si-NC, si-MALAT1-1, si-MALAT1-2, or si-MALAT1-3, respectively. And the expression of MALAT1 in transfected cells was assayed by qRT-PCR. (C-H) MPP+-stimulated SK-N-SH and SK-N-BE cells were transfected with si-NC or si-MALAT1-1, respectively. **c**, **d** After transfection for 0, 24, 48, or 72 h, cell viability was assessed using MTT assay. **e** The apoptosis rate of transfected cells was analyzed by flow cytometry assay. **f**, **g** Western blot assay was performed to determine the levels of apoptosis-related protein Bax, Cleaved-casp-3, and Bcl-2. **h** The protein level of PCNA was detected by western blot assay. The experiments were repeated three times (n = 3). *P < 0.05, compared to si-NC group

To determine the role of MALAT1 in PD, loss-of-function experiments were performed by transfecting si-NC or si-MALAT1 in MPP^+^-stimulated SK-N-SH and SK-N-BE cells. As shown in Fig. 2a and b, MALAT1 expression was reduced by half in cells with si-MALAT1-1, si-MALAT1-2, or si-MALAT1-3 transfection compared to si-NC group. The knockdown efficiency of si-MALAT1-1 was more distinctly than the others, hence, si-MALAT1-1 was chosen for further study. MTT assay indicated that downregulation of MALAT1 increased cell viability in contrast with the cells with si-NC transfected group in MPP^+^-stimulated SK-N-SH and SK-N-BE cells (Fig. 2c and d). Besides, flow cytometry assay was used to determine cell apoptosis rate, and the results indicated that cell apoptosis was remarkably curbed by MALAT1 depletion (Fig. 2e). And it was further confirmed by the decreased protein levels of Bax (pro-apoptotic protein) and Cleaved-casp-3 (marker for apoptosis) and the elevated level of Bcl-2 (anti-apoptotic protein) protein in cells with MALAT1 downregulation (Fig. 2f and g). Besides, we also evaluated the protein level of PCNA (cell division marker) in transfected cells. As shown in Fig. 2h, the protein level of PCNA was doubled in cells with MALAT1 knockdown, explaining the increased cell viability in cells with si-MALAT1 transfection. Thus, downregulation of MALAT1 promoted cell proliferation but inhibited cell apoptosis in MPP^+^-stimulated SK-N-SH and SK-N-BE cells.

### MALAT1 acted as a miRNA sponge for miR-135b-5p

**Fig. 3:**
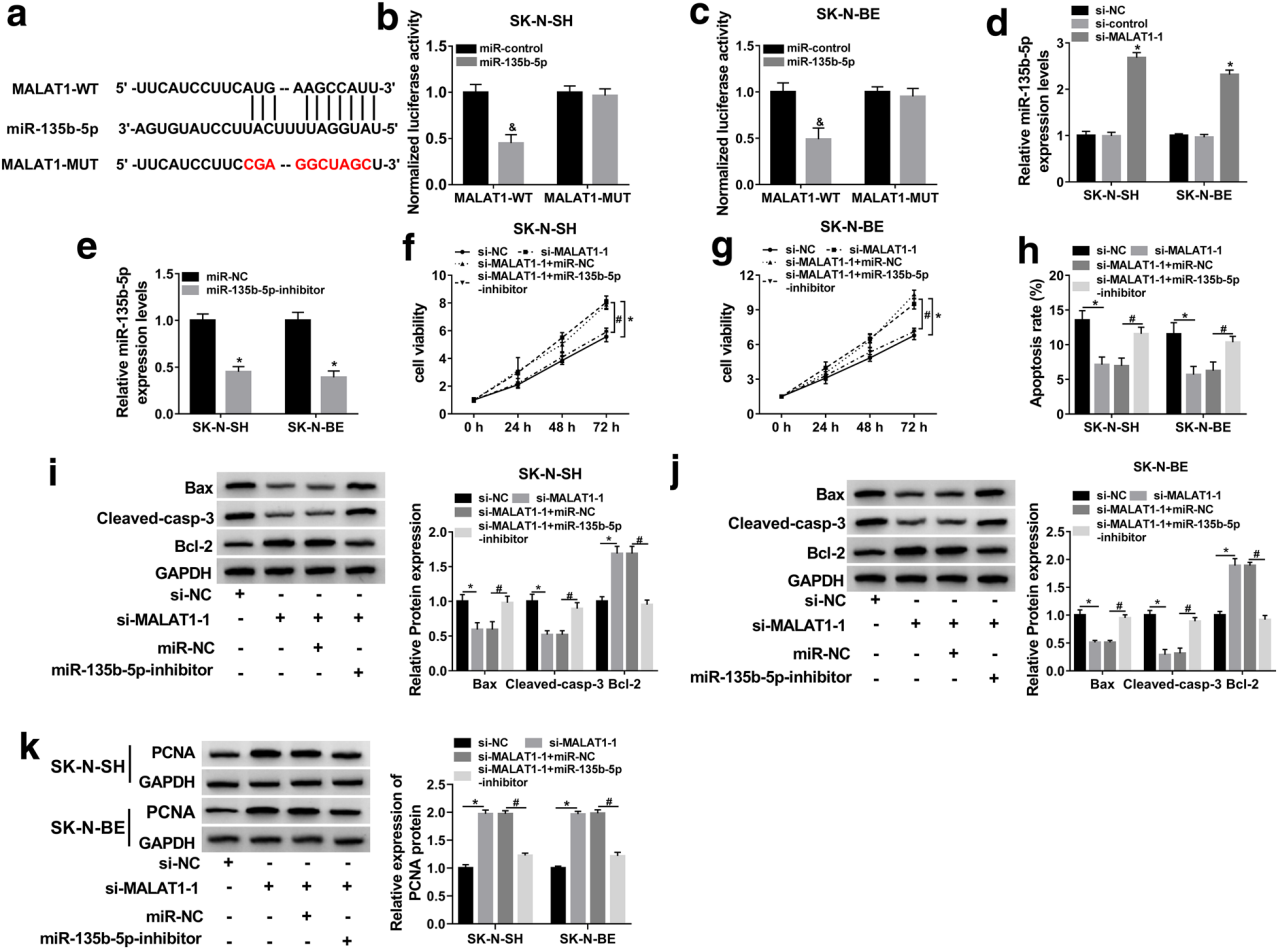
MALAT1 acted as a miRNA sponge of miR-135b-5p. **a** StarbaseV3.0 was used to verify the binding sites between miR-135b-5p and MALAT1, and MALAT1 sequences contained wild type (MALAT1-WT) or mutant type (MALAT1-MUT) miR-135b-5p binding sites were shown. **b**, **c** Dual-luciferase reporter assay was conducted to evaluate the interrelation between miR-135b-5p and MALAT1 in MPP^+^-treated SK-N-SH and SK-N-BE cells. **d** The effect of si-MALAT1 on miR-135b-5p expression was confirmed by qRT-PCR assay. **e** The inhibitory efficiency of miR-135b-5p inhibitor on miR-135b-5p level was examined via qRT-PCR assay. **f**, **g** MTT assay was employed to detect the roles of si-MALAT1 and miR-135-5p inhibitor in cell proliferation. **h** Apoptosis rates of MPP^+^-induced SK-N-SH and SK-N-BE cells were detected using flow cytometry analysis. **i**, **j** The protein levels of Bax, Cleaved-casp-3, and Bcl-2 were detected by western blot assay. **k** The protein level of PCNA in transfected cells was detected by western blot. The experiments were repeated three times (n = 3). *P < 0.05 compared to si-NC group, ^&^*P* < 0.05 compared to miR-control group, ^#^*P* < 0.05 compared to si-MALAT1 + miR-NC

To investigate the mechanism underlying MALAT1 in PD, the bioinformatics tool StarbaseV3.0 was used to predict the target miRNAs of MALAT1. As shown in Fig. 3a, MALAT1 harbored complementary binding sites of miR-135b-5p. To verify whether MALAT1 was interacted with miR-135b-5p, dual-luciferase reporter assay was employed. Compared with the miR-control transfected group, transfection of miR-135b-5p obviously inhibited the luciferase activity of MALAT1-WT group, while it had little effect on the luciferase activity of MALAT1-MUT group (Fig. 3b, c), demonstrating their potential endogenous interaction in SK-N-SH and SK-N-BE cells. In addition, miR-135b-5p expression was remarkably increased by MALAT1 knockdown (Fig. 3d), and was markedly decreased by the miR-135b-5p inhibitor (Fig. 3e). Next, to investigate whether MALAT1 regulated cell behaviors by miR-135b-5p, SK-N-SH and SK-N-BE cells were transfected with si-NC, si-MALAT1, si-MALAT1 + miR-NC or si-MALAT1 + miR-135b-5p-inhibitor, respectively. We found that miR-135b-5p inhibitor reversed the promotion effect of si-MALAT1 on cell viability (Fig. 3f and g). And miR-135b-5p inhibitor rescued cell apoptosis that was repressed by MALAT1 knockdown, as the apoptosis rate was decreased by si-MALAT1, but was increased by co-transfection of miR-135b-5p inhibitor (Fig. 3h). And si-MALT1-mediated the suppression on the protein levels of Bax and Cleaved-casp-3, and the promotion on Bcl-2 protein, were reversed by miR-135b-5p inhibitor (Fig. 3i, j). Besides, the elevated protein level of PCNA that induced by si-MALAT1 was reversed by miR-135b-5p inhibitor (Fig. 3k). These results indicated that MALAT1 knockdown regulated proliferation and apoptosis of MPP+-stimulated SK-N-SH and SK-N-BE cells via regulating miR-135b-5p.

### MiR-135b-5p directly targeted GPNMB

**Fig. 4:**
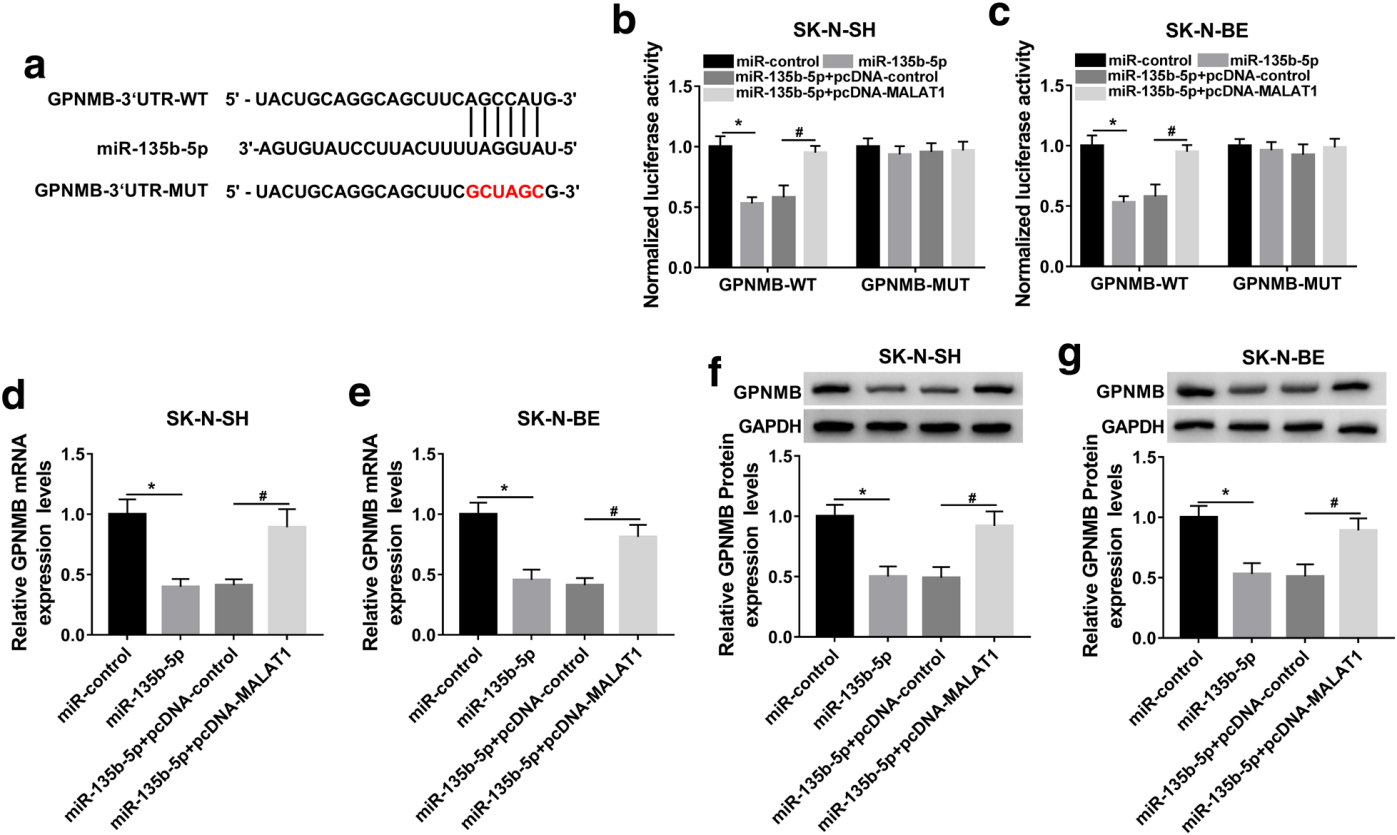
GPNMB was a target of miR-135-5p. **a** The binding sites between miR-135b-5p and GPNMB was predicted by StarbaseV3.0 software. **b–d** Cells with GPNMB-WT or GPNMB-MUT transfection were transfected with miR-control, miR-135b-5p, miR-135b-5p + pcDNA-control, miR-135b-5p + pcDNA-MALAT1, respectively. **b** and **c** Relative luciferase activity was assayed by dual-luciferase reporter assay. **d** and **e** QRT-PCR was employed to estimate the impacts of miR-135b-5p mimic and pcDNA-MALAT1 on the mRNA level of GPNMB. **f** and **g** The protein level of GPNMB was evaluated via western blot assay. The experiments were repeated three times (n = 3). *P < 0.05 compared to miR-control, ^#^*P* < 0.05 compared to miR-135b-5p + pcDNA-control

To further explore the underlying mechanism of miR-135b-5p in PD, StarBase3.0 software was applied to predict the potential target of miR-135b-5p, and the results revealed that GPNMB might be target by miR-135b-5p (Fig. 4a). Subsequently, dual-luciferase reporter assay displayed that miR-135b-5p could significantly lower the luciferase activity of GPNMB-WT group without significant influence on the luciferase activity of GPNMB-MUT. However, this inhibitory effect of GPNMB-WT and miR-135b-5p mimic on luciferase activity was rescued by co-transfection of pcDNA-MALAT1 (Fig. 4b, c), indicating the potential endogenous interaction in SK-N-SH and SK-N-BE cells. To investigate whether GPNMB was regulated by miR-135b-5p and MALAT1, miR-control, miR-135b-5p, miR-135b-5p + pcDNA-control, or miR-135b-5p + pcDNA-MALAT1 was transfected into MPP^+^-induced SK-N-SH and SK-N-BE cells, qRT-PCR assay implied that miR-135b-5p mimic obviously inhibited mRNA and protein levels of GPNMB, while these inhibiting effects were reversed by transfection of pcDNA-MALAT1 (Fig. 4d and g). The results implicated that MALAT1 regulated GPNMB expression by sponging miR-135b-5p.

Upregulation of GPNMB abolished the effect of MALAT1 knockdown on cell proliferation and apoptosis in MPP^+^-stimulated SK-N-SH and SK-N-BE cells

**Fig. 5:**
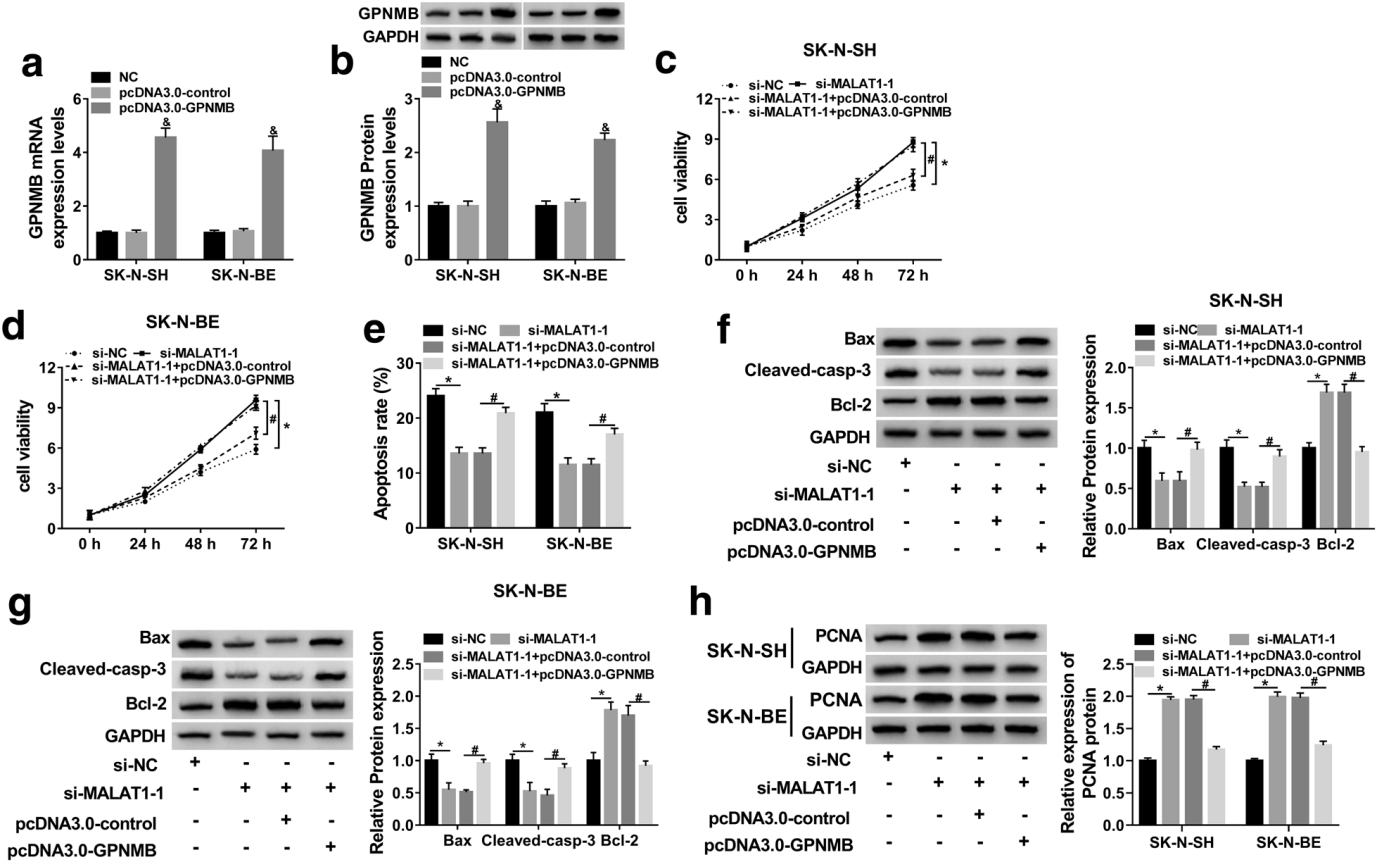
Overexpression of GPNMB reversed the effect of MALAT1 depletion on cell proliferation and apoptosis in MPP^+^-stimulated SK-N-SH and SK-N-BE cells. **a**, **b** MPP^+^-treated SK-N-SH and SK-N-BE cells were transfected with pcDNA3.0-control or pcDNA3.0-GPNMB for 48 h. **a** The mRNA level of GPNMB was determined by qRT-PCR. **b** GPNMB protein level was assessed by western blot assay. **c**–**h** MPP^+^-treated SK-N-SH and SK-N-BE cells were transfected with si-NC, si-MALAT1-1, si-MALAT1-1 + pcDNA3.0-control, or si-MALAT1-1 + pcDNA3.0-GPNMB, respectively. **c**, **d** Cell viability was detected by MTT assay. **e** Flow cytometry assay was conducted to identify cell apoptosis *in vitro*. **f**, **g** Western blot assay was used to evaluate the expression levels of apoptosis-related proteins, including Bax, Cleaved-casp-3, and Bcl-2. **h** The protein level of PCNA was detected by western blot. The experiments were repeated three times (n = 3). *P < 0.05 compared to si-NC, ^&^*P* < 0.05 compared to pcDNA3.0-control, ^#^*P* < 0.05 compared to si-MALAT1 + pcDNA-control

Then, the regulatory mechanism of GPNMB in PD was further confirmed. Firstly, the over-expressed efficiency of pcDNA3.0-GPNMB was confirmed by qRT-PCR and western blot assay (Fig. 5a and b). And MTT assay revealed that upregulation of GPNMB reversed the promotion effect of MALAT1 knockdown on cell viability (Fig. 5c and d). Besides, the effect of MALAT1 knockdown on cell apoptosis was also reversed by GPNMB upregulation (Fig. 5e–g). Furthermore, overexpression of GPNMB also reversed the elevated protein level of PCNA that mediated by si-MALAT1, indicating that upregulation of GPNMB promoted cell proliferation of MPP+-stimulated SK-N-SH and SK-N-BE cells (Fig. 5h). Thus, MALAT1 could regulate cell proliferation and apoptosis by miR-135b-5p/GPNMB aixs in MPP^+^-stimulated SK-N-SH and SK-N-BE cells.

## Discussion

PD is a neurological disorder that affects the movement abilities of adults [1, 2], which remains lack of effective therapeutic measures due to the limited understanding of the dysfunction of molecular events and signaling pathways underlying neurodegeneration [6]. In the present research, MPP^+^, the active metabolite of MPTP, was used to stimulate SK-N-BE and SK-N-BE cells, thereby established PD cell model according to previous descriptions [30, 31]. We found that MALAT1 was highly expressed in MPP^+^-induced SK-N-BE and SK-N-BE cells. Meanwhile, cell viability was notably decreased in SK-N-BE and SK-N-BE cells with the increasing concentration of MPP^+^.

It has been reported that MALAT1 was highly expressed in neurons, and the aberrant expression of MALAT1 could regulate target gene expression associated with synapse formation [32]. Besides, Liu et al. indicated that MALAT1 promoted the apoptosis by sponging miR-124 in mouse models of PD and *in vitro* model of PD [33]. Cai et al. showed that MALAT1 could induce inflammasome activation and reactive oxygen species (ROS) production in PD mouse and microglial cell models by epigenetically inhibiting NRF2 [34]. Consistent with previous research, in our study, MALAT1 depletion rescued the decreased cell proliferation and increased apoptosis in MPP^+^-treated SK-N-BE and SK-N-BE cells, indicating the pivotal role of MALAT1 in the apoptosis of neurons in PD.

Extensive research suggested that lncRNAs could act as the molecular sponge of miRNAs to negatively regulate its expression and thus regulating biological functions [35]. In this study, we firstly found that miR-135b-5p was a target miRNA of MALAT1. Sufficient evidence suggested that miR-135b-5p exhibited multiple biological functions in human disease [36–38]. Ren et al. indicated that miR-135b-5p regulated the proliferation and apoptosis of ovarian cancer [36]. Zhang et al. suggested that miR-135b-5p promoted cells migration, invasion and EMT in pancreatic cancer cells [37]. Besides, Huang et al. indicated that miR-135b-5p could reduce neuronal injury and inflammatory response in post-stroke cognitive impairment [38]. A recent research also showed that miR-135b-5p was aberrantly expressed in dopaminergic neurons of PD patients, whereas the research of miR-135b-5p functional effect in PD was limited [39]. In our research, downregulation of miR-135b-5p partly reversed the elevated cell proliferation and decreasing cell apoptosis which was mediated by MALAT1 knockdown, indicating the protect role of miR-135b-5p in MPP^+^-stimulated SK-N-SH and SK-N-BE cells.

MiRNAs could regulate expression of genes at post-transcriptional level by binding 3’UTR of mRNA. Tumor suppressor gene adenomatous polyposis coli (APC) [40], oncogene forkhead box O1 (FOXO1) [41], Anterior Gradient 2 (AGR2) [42] and cyclin-associated protein cyclin D2 [43] were reported as the target genes of miR-135b-5p. In our research, we found that miR-135b-5p was interacted with GPNMB, and the interaction between miR-135b-5p and GPNMB was further confirmed by subsequent dual-luciferase reporter assay. It was reported that GPNMB was abnormal expressed in various cells [24, 44]. Besides, genome-wide association studies demonstrated that GPNMB should be considered as possible Parkinson’s disease-related genes for future studies [45]. Thus, we further investigate the effect of GPNMB in MALAT1/miR-135b-5p regulated the proliferation and apoptosis of neurons in PD. In the present study, we revealed that MALAT1 upregulated GPNMB protein level by inhibiting miR-135b-5p, thereby regulating cell proliferation and apoptosis in MPP^+^-stimulated SK-N-SH and SK-N-BE cells.

## Conclusions

**Fig. 6:**
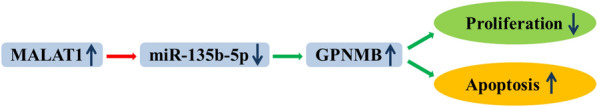
The schematic Figure presented that the MALAT1/miR-135b-5p/GPNMB axis

Taken together, MALAT1 expression was increased in MPP^+^-induced SK-N-BE and SK-N-BE cells. Knockdown of MALAT1 promoted cell proliferation, and hamper apoptosis in MPP^+^-induced SK-N-SH and SK-N-BE cells. MALAT1 interacted with miR-135b-5p to regulate GPNMB expression (Fig. 6). Our results highlight the potential of MALAT1/miR-135b-5p/GPNMB network in PD cell model, and provide a potential therapeutic target for PD. However, more efforts should be given to elaborate the regulatory network of lncRNA/miRNA/mRNA as central player in PD.

## Supplementary Information

**Additional file 1: Figure S1.** The levels of TUJ1 and VMAT2 during the cells with MPP+ treatment. Cells were treated with 2mM MPP+ for 6 days, and the expression of TUJ1 and VMAT2 were detected by qRT-PCR.

## Data Availability

All data generated and analysed during this study are included in this published article are available on request.
