# Supplementary figures and images for: Long non‐coding RNA MALAT1 regulates cell proliferation and apoptosis via miR-135b-5p/GPNMB axis in Parkinson’s disease cell model
Source: Biol Res. 2021 Mar 16;54:10. doi: 10.1186/s40659-021-00332-8 (PMC7968316; doi:10.1186/s40659-021-00332-8)

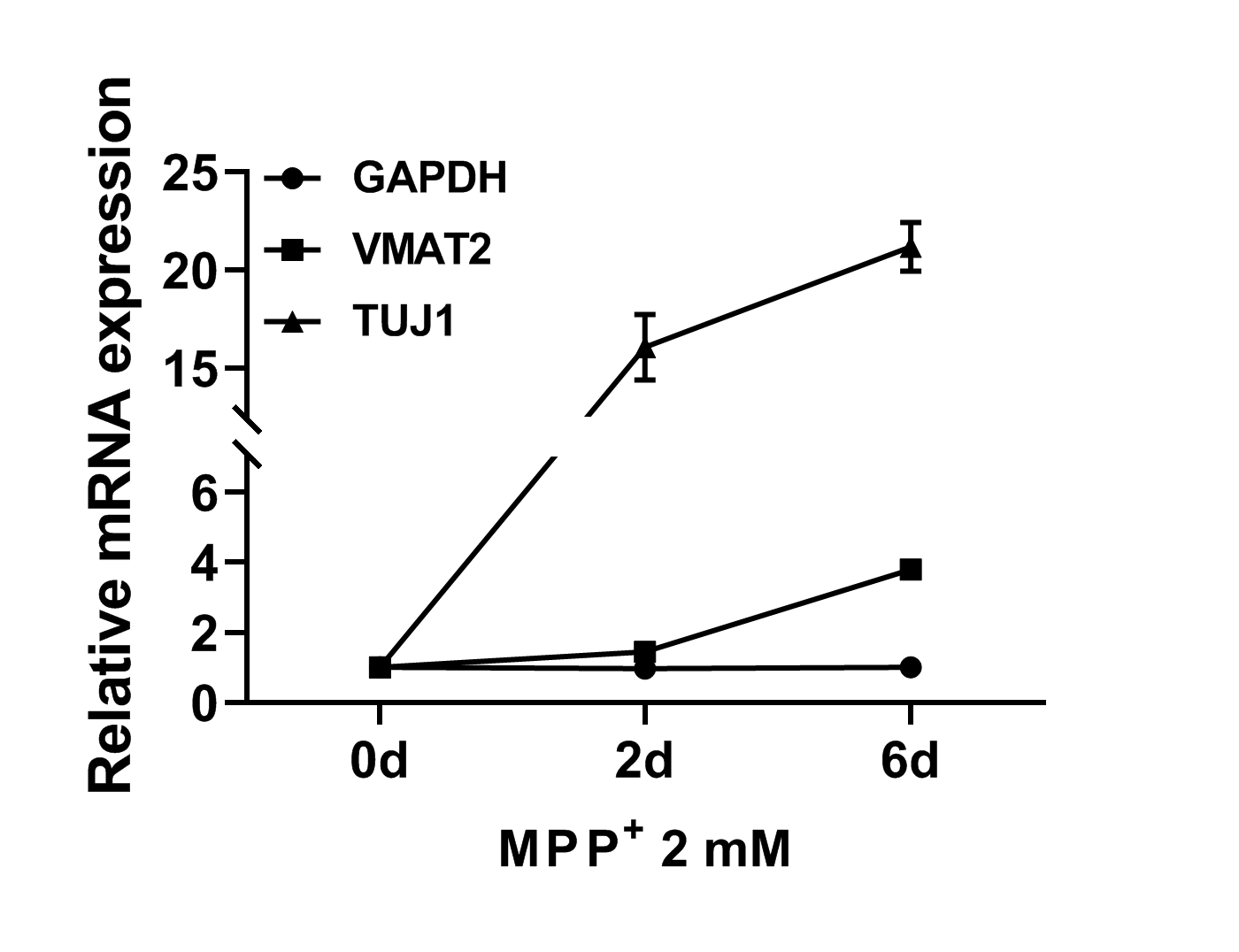

Supplement: Supplementary file 1 — Additional file 1: Figure S1. The levels of TUJ1 and VMAT2 during the cells with MPP+ treatment. Cells were treated with 2mM MPP+ for 6 days, and the expression of TUJ1 and VMAT2 were detected by qRT-PCR. [file 40659_2021_332_MOESM1_ESM.tif]
